# Efficacy and Safety of Fundoplication Sleeve Gastrectomy in Obesity and GERD: A Systematic Review and Meta-Analysis

**DOI:** 10.3390/jcm14217723

**Published:** 2025-10-30

**Authors:** Filipe Amorim-Cruz, Diogo Fernandes Lopes, Bernardo Sousa-Pinto, Hugo Santos-Sousa

**Affiliations:** 1 Faculty of Medicine, University of Porto—Alameda Prof. Hernâni Monteiro, 4200-319 Porto, Portugal; 2 Surgery Department, São João University Medical Center, Alameda Prof. Hernâni Monteiro, 4200-319 Porto, Portugal; 3 MEDCIDS—Department of Community Medicine, Information and Health Decision Sciences, Faculty of Medicine, Rua Dr. Plácido da Costa, 4200-450 Porto, Portugal; 4 CINTESIS—Center for Health Technologies and Services Research, University of Porto, Rua Dr. Plácido da Costa, 4200-450 Porto, Portugal; 5 Obesity Integrated Responsibility Unit (CRI-O), São João University Medical Center, Alameda Prof. Hernâni Monteiro, 4200-319 Porto, Portugal

**Keywords:** GERD, fundoplication, sleeve gastrectomy

## Abstract

**Background/Objectives**: Laparoscopic sleeve gastrectomy (SG) is the most performed bariatric procedure, providing effective weight loss and comorbidity improvement. However, its association with new-onset or worsening gastroesophageal reflux disease (GERD) remains a limitation. To address this, fundoplication sleeve gastrectomy (FSG) has been proposed by combining SG with an anti-reflux procedure. This systematic review and meta-analysis evaluates the efficacy and safety of FSG in patients with severe obesity. **Methods**: PubMed, Scopus, and Web of Science were systematically searched up to December 2023. Eligible studies included adults with BMI ≥ 40 kg/m^2^ or ≥35 kg/m^2^ with comorbidities undergoing FSG or SG with ≥12 months of follow-up. Random-effects meta-analysis compared FSG and SG in terms of weight loss, postoperative GERD, and complications. **Results:** Twelve studies (n = 543) were included; five contributed to the meta-analysis. Pooled analysis showed no significant difference in percentage of excess weight loss (%EWL) between FSG and SG (Hedges’ g = −0.11; 95% CI: −0.99–0.76; I^2^ = 86%), and similar %TWL outcomes (Hedges’ g = −0.28; 95% CI: −0.70–0.13). FSG demonstrated a significantly lower postoperative GERD prevalence (RR = 0.08; 95% CI: 0.01–0.47) and greater GERD resolution (RR = 1.86; 95% CI: 0.80–4.20), but higher complication (RR = 2.95; 95% CI: 1.02–8.50) and reoperation rates (RR = 4.39; 95% CI: 1.47–13.12). **Conclusions**: FSG achieves weight loss comparable to SG and may reduce postoperative GERD prevalence, but carries an increased complication and reoperation risk. Further randomized trials with standardized GERD definitions and longer follow-up are required.

## 1. Introduction

Over the past decade, laparoscopic sleeve gastrectomy (SG) has become the most frequently performed bariatric procedure worldwide, largely due to its high efficacy, low complication rate, and relative technical simplicity compared to malabsorptive procedures [1,2]. Randomized trials, such as SM-BOSS and SLEEVEPASS, demonstrate that SG provides weight loss and comorbidity outcomes comparable to Roux-en-Y Gastric Bypass (RYGB), with a good long-term quality of life [3,4]. Based on the literature, the percentage of excess weight loss (%EWL) after SG typically exceeds 50% within 1–5 years, and significant improvements in type 2 diabetes, hypertension, and dyslipidemia are consistently reported, all with a low perioperative morbidity and mortality rate. Major complications such as leaks (1–2%), bleeding, or strictures are relatively infrequent and tend to decrease with surgical experience, underscoring SG’s favorable safety profile [5].

Despite these advantages, the main long-term drawback of SG is the development or worsening of gastroesophageal reflux disease (GERD), with reported symptom prevalence ranging from 20% to 60% following the procedure [6,7,8], accompanied by higher rates of erosive esophagitis and Barrett’s esophagus [5,9]. The higher rate of pre-existing or “de novo” GERD after SG is complex and multifactorial. On one hand, preoperatively, obese patients have a higher tendency to GERD due to intra-abdominal adiposity compression, consequently leading to an altered gastroesophageal pressure. On the other hand, “de novo” GERD could be explained based on increased intraluminal gastric tube pressure, division of sling fibers, and damage to the angle of His, which leads to hypotony of the lower esophageal sphincter and gastric dysmotility [10,11]. Given the rising concerns about long-term esophageal complications, including Barrett’s esophagus and adenocarcinoma, careful patient selection and surveillance strategies for SG have been recommended.

Based on the International Federation for the Surgery of Obesity and Metabolic Disorders (IFSO) guidelines [12], RYGB is often recommended as the preferred surgical option for patients with severe obesity and preexisting GERD or hiatal hernia [13]. However, its efficacy is not absolute. A Swedish nationwide cohort study of over 4200 patients found that reflux symptoms persisted or recurred in approximately 50% of patients up to 10 years after RYGB [14,15]. Moreover, RYGB carries its own long-term complications, including internal hernias, marginal ulcers, and nutritional deficiencies [16].

Therefore, to reduce the risk of GERD symptoms after SG and broaden the applicability of SG in patients with mild reflux, several technical modifications have been explored, including anti-reflux sleeve gastroplasty [17], fundoplication sleeve gastrectomy (FSG) [11,18,19], SG combined with the LINX system [20], and SG with crural repair [21].

Some of these techniques, such as crural repair, have shown conflicting results regarding the reduction in GERD after SG, while FSG has been shown to be a promising modification of SG, since it integrates a better anti-reflux mechanism. By combining SG with a concomitant fundoplication (Nissen, Rossetti, Dor, or Toupet), FSG aims to restore the anti-reflux barrier, protect the staple line, prevent postoperative GERD, and offer a safe and effective alternative for patients in whom RYGB is contraindicated [22]. Recent studies have reported encouraging outcomes. Randomized and comparative trials have shown that FSG achieves effective weight loss while potentially reducing GERD prevalence compared with standard SG [21,23]. Likewise, recent systematic reviews and meta-analyses suggest that FSG provides satisfactory %EWL and lower GERD recurrence rates, although results vary depending on the type of fundoplication used [24,25,26].

Nevertheless, several uncertainties remain. Evidence is still limited by heterogeneity in surgical techniques (Nissen vs. Toupet vs. Dor fundoplication), lack of long-term follow-up, and inconsistent diagnostic criteria for GERD [27,28]. Furthermore, safety concerns have been raised, as integrating fundoplication into SG adds technical complexity and may increase the risk of complications or reoperations [9].

Given these limitations, a comprehensive synthesis of the available evidence is required. This systematic review and meta-analysis therefore aimed to evaluate the effectiveness, safety, and feasibility of FSG compared with standard SG in patients with severe obesity.

## 2. Materials and Methods

This systematic review with meta-analysis follows the Preferred Reporting Items for Systematic Reviews and Meta-analyses (PRISMA) statement guidelines (Table S1) and the recommendations of the Cochrane Handbook for Systematic Reviews [29,30], though it was not prospectively registered in an international registry. However, all methodological steps—including the search strategy, inclusion/exclusion criteria, and planned analyses—were defined before study initiation (protocol developed and timestamped in September 2023; see Supplementary Material).

Search strategy: We searched PubMed, Scopus, and Web of Science until 31 December 2023 (when our search was performed). Search queries are detailed in Table 1. No restrictions were set regarding language or publication year. Any disagreements were solved by a third author (H.S.S).

Study Selection and Eligibility Criteria: After removing duplicates, each study was independently assessed by two reviewers (F.A.C. and D.F.L.), first by title and abstract screening and then by full-text reading. We included original studies assessing FSG in patients with a body mass index (BMI) of ≥40 kg/m^2^ or ≥35 kg/m^2^ with obesity comorbidities. The primary outcome was the postoperative prevalence and resolution of GERD, weight loss parameters, and postoperative overall complications. The exclusion criteria comprised studies with a follow-up duration of less than 1 year, studies that were reviews, meta-analyses, case reports, technical reports, editorials, letters to the editor, and animal studies.

Data Extraction: Data were independently extracted by two authors (F.A.C. and D.F.L.) into a predesigned data extraction form, developed according to the Cochrane Handbook [29]. The extracted data included author identification, year of publication, country, study design, patients’ inclusion and exclusion criteria, general characteristics of the participants (age, gender, and mean preoperative body mass index), type of FSG, mean operative time, and mean hospital length of stay. Postoperative gastroesophageal reflux disease (GERD) was considered a primary outcome and defined according to the Montreal Consensus, encompassing both symptom-based and objectively confirmed reflux [31]. For each included study, GERD diagnosis methods were recorded and categorized as: (1) symptom-based (validated questionnaires or clinical diagnosis), (2) endoscopic-based (presence of erosive esophagitis or Barrett’s esophagus), and (3) functional-based (objective measurements via 24 h pH monitoring or esophageal manometry). When multiple assessments were available, objective criteria were prioritized. Progress of weight loss was defined as the %EWL and percentage of total weight loss (%TWL), based on these formulas: %EWL = [(Initial weight) − (Postoperative weight)]/[(Initial weight) − ideal weight] × 100, with an ideal weight corresponding to a BMI of 25 kg/m^2^; %TWL = [(Initial weight) − (Postoperative weight)]/(Initial weight). Postoperative complications were classified according to the Clavien–Dindo system [32], with overall complications defined as events of grade III or higher. When available, complications were further categorized as early (≤30 days) or late (>30 days) to allow subgroup comparisons. Reported adverse events included bleeding, staple line leaks, technical issues related to the fundoplication (e.g., wrap tightness, ischemia, or migration), and reoperations, the latter mainly performed for weight regain or persistent reflux.

Quality Assessment: The risk of bias for each included study was independently assessed by two reviewers (F.A.C. and D.F.L.). For observational studies, we used both the National Institutes of Health (NIH) quality assessment tool and the ROBINS-I (Risk of Bias in Non-randomized Studies of Interventions) tool to ensure a comprehensive evaluation of methodological quality across key domains (confounding, selection, classification of interventions, deviations from intended interventions, missing data, measurement of outcomes, and selection of reported results). For the randomized controlled trial, the Cochrane RoB 2 tool was applied, assessing bias in randomization, deviations from intended interventions, missing outcome data, measurement of outcomes, and reporting. Discrepancies were resolved by consensus [33,34,35].

Quantitative Synthesis of Results: We performed a random-effects meta-analysis for both continuous and categorical variables, using the DerSimonian–Laird method for categorical outcomes and standardized mean differences (SMD) with Hedges’ g correction for continuous outcomes. For categorical variables (postoperative GERD and postoperative complications), we calculated pooled risk ratios (RR) with 95% confidence intervals (CI). For continuous variables (%EWL), pooled SMDs with Hedges’ g correction, which accounts for small-sample bias, along with 95% CI. The postoperative prevalence of GERD was extracted as the proportion of patients diagnosed with GERD in each intervention group (FSG Vs. SG) at the longest available follow-up, provided the minimum follow-up duration was ≥12 months.

Meta-analysis was conducted only when a variable of interest (postoperative prevalence of GERD symptoms, weight loss parameters, or postoperative complications) was reported in at least two primary studies comparing FSG and SG, and studies were sufficiently comparable in population, intervention, and outcome definitions. When only two studies were available, pooling was performed only if their results were methodologically and clinically consistent. Statistical heterogeneity was evaluated using the Cochran’s Q test and the I^2^ statistic. A *p*-value < 0.10 on Cochran’s Q test was considered indicative of significant heterogeneity. For the I^2^ statistic, values of 0% were interpreted as no heterogeneity, 0–10% as low, 10–50% as moderate, and >50% as high heterogeneity [36]. Leave-one-out sensitivity analyses were performed to examine potential sources of heterogeneity visually. Given the limited number of eligible studies (k = 3–4), formal meta-regression was not feasible. Instead, exploratory stratified forest plots were generated. For all outcomes (postoperative GERD prevalence, FSG effectiveness, and associated complications), potential sources of heterogeneity—such as follow-up duration (12 months vs. ≥24 months)—were considered. Exploratory subgroup analysis by the type of fundoplication was planned but could not be performed because each primary study employed a different fundoplication technique (Rossetti, Toupet, or Nissen). In the analysis of postoperative GERD prevalence, additional sensitivity analyses were conducted, stratified by the method of GERD assessment (symptom-, endoscopy-, or function-based).

Publication bias was not formally assessed using funnel plots or Egger’s regression test, as each meta-analysis included fewer than 10 studies, rendering these methods statistically unreliable according to Cochrane guidance [29]. To explore potential small-study effects, Copas’ selection model was applied as a sensitivity analysis to examine how varying degrees of publication bias could influence the pooled estimates.

All analyses were conducted using R statistical software (version 4.4.1; R Foundation for Statistical Computing, Vienna, Austria), employing the “meta” package [37] for the DerSimonian–Laird and Hedges’ g models.

## 3. Results

### 3.1. Study Selection

**Figure 1:**
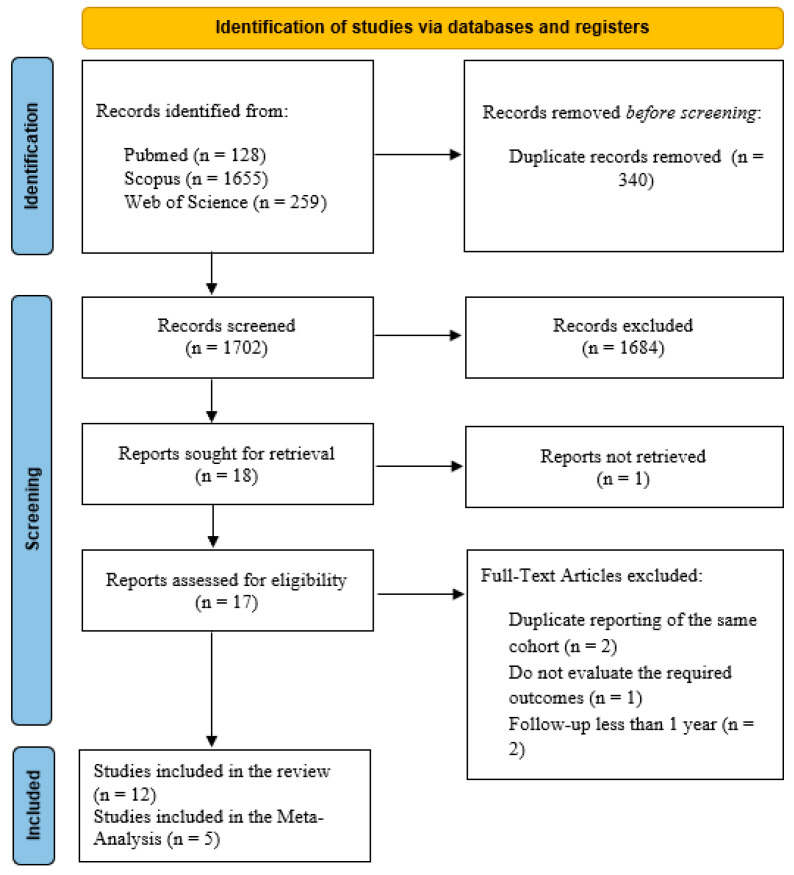
Flow diagram of study selection.

Figure 1 displays the PRISMA diagram of our search process. After searching three databases, we identified 2042 articles, with 340 being duplicates. Following the screening process, 18 articles were selected for full-text review. One author was contacted for additional information about missing outcomes of interest, but as the author did not respond, the study was excluded. In the end, 12 studies were included in the qualitative synthesis, and 5 in the quantitative synthesis.

### 3.2. Study Characteristics

Table 2 presents a summary of the characteristics of the studies included in this systematic review. Of the 12 studies included, 6 were retrospective observational studies [9,38,39,40,41,42], 5 were prospective observational studies [11,18,19,43], and one was a randomized controlled trial [44]. A total of 543 patients were studied, with the sample size ranging from 28 to 365, and the follow-up period ranged from 12 months to 108 months. Fundoplication technique varied between studies and included three types of FSG—anterior (Dor), posterior (Toupet), and total (Nissen or Rossetti).

### 3.3. Effectiveness of FSG—Weight Loss

**Figure 2:**
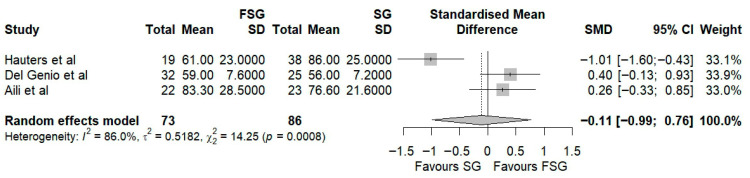
Forest plot representing the pooled standardized mean difference in %EWL between FSG and SG. Each square represents an individual study’s effect estimate with its 95% confidence interval (horizontal line). The size of the square reflects the study’s weight in the meta-analysis. The dashed vertical line indicates the line of no effect (SMD = 0), and the diamond (shadowed area) represents the pooled effect estimate with its 95% confidence interval [9,11,41].

**Figure 3:**
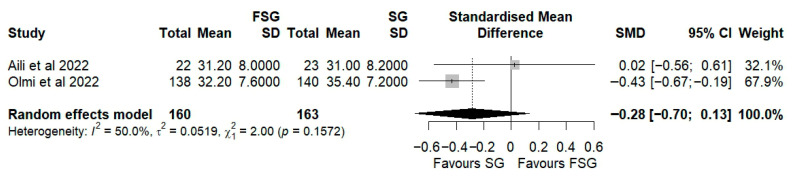
Forest plot representing the pooled standardized mean difference in %TWL between FSG and SG. Each square represents an individual study’s effect estimate with its 95% confidence interval (horizontal line). The size of the square reflects the study’s weight in the meta-analysis. The dashed vertical line indicates the line of no effect (SMD = 0), and the diamond (shadowed area) represents the pooled effect estimate with its 95% confidence interval [9,45].

In our systematic review, twelve studies encompassing 543 patients evaluated the impact of FSG on weight loss using %EWL, %TWL, or %EBMIL as primary anthropometric outcomes. Among those reporting %EWL, eight studies provided data at 12 months of follow-up. At 12 months, %EWL ranged from 58 ± 23% to 83.3 ± 28.5% (Table 3), indicating satisfactory postoperative weight loss consistent with Reinhold’s success criteria. In the pooled meta-analysis of three studies (Figure 2, the standardized mean difference (Hedges’ g = −0.11; 95% CI: −0.99 to 0.76) slightly favored SG, though not significantly. Substantial heterogeneity was observed (I^2^ = 86%), suggesting methodological and clinical variability across studies. To explore this variability, an exploratory stratified forest plot was constructed by follow-up duration (Figure S1). At 12 months, the pooled SMD was −0.11 (95% CI: −0.99 to 0.76; I^2^ = 86%), and at 24 months, −0.24 (95% CI: −1.26 to 0.78; I^2^ = 83.8%). The test for subgroup differences was not statistically significant (*p* = 0.85), indicating no consistent effect modification by follow-up time. Subgroup exploration by fundoplication type was not feasible, as each included study employed a distinct wrap configuration (Rossetti, Toupet, or Nissen). More recently, IFSO guidelines [46] recommended %TWL as the preferred metric for evaluating postoperative weight loss after metabolic bariatric surgery. Therefore, we also analyzed the %TWL in four studies at 12 months of follow-up, ranging from 25 ± 8% to 32.2 ± 7.6% (Table 3). The pooled analysis of two studies again showed no significant difference between FSG and SG (Hedges’ g = −0.28; 95% CI: −0.70 to 0.13; Figure 3).

To assess potential publication bias (Table S2), the adjusted effect estimate (−0.11; 95% CI: −0.98 to 0.76) was nearly identical to the unadjusted estimate, and the residual selection bias test (*p* = 0.29) indicated no statistically significant bias. These results suggest that small-study effects or selective publication are unlikely to have influenced the pooled estimate.

Taken together, these findings suggest that FSG achieves weight loss outcomes comparable to standard SG, confirming its metabolic efficacy. However, the considerable variability across studies underscores the need for standardized reporting and longer-term follow-up to clarify whether subtle differences in technique or patient selection may influence sustained weight reduction.

### 3.4. Postoperative GERD Outcomes

#### 3.4.1. Postoperative GERD Prevalence

**Figure 4:**
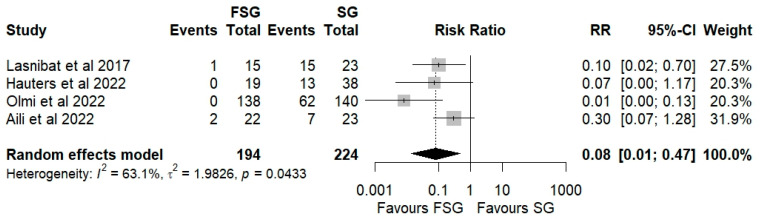
Forest plot showing the risk ratio of postoperative prevalence of GERD between FSG and SG. Each square represents an individual study’s effect estimate with its 95% confidence interval (horizontal line). The size of the square reflects the study’s weight in the meta-analysis. The dashed vertical line indicates the line of no effect (RR = 1), and the diamond (shadowed area) represents the pooled effect estimate with its 95% confidence interval [9,39,41,45].

Four studies, including a total of 418 patients, compared postoperative GERD prevalence between FSG and SG. The pooled analysis demonstrates that FSG is associated with a substantially lower postoperative prevalence of GERD compared to SG, with a pooled RR of 0.08 (95% CI: 0.01–0.47; *p* < 0.05, Figure 4). This effect size is consistent across studies, with all individual RRs favoring FSG (ranging from 0.01 to 0.30); however, the confidence intervals are wide, reflecting variability in study size and measurement precision. The between-study heterogeneity is moderate (I^2^ = 63.1%, Q Cochran *p*-value = 0.0433), suggesting that part of the variability in effect estimates may be due to differences in study populations, diagnostic criteria for GERD, or surgical techniques (e.g., type of fundoplication).

Subgroup analyses were performed based on the method of GERD ascertainment (Figure S2) and by follow-up duration (Figure S3). When GERD was identified by symptom-based assessment, the pooled RR was 0.33 (95% CI: 0.13–0.81; I^2^ = 41%). In studies that employed endoscopic evaluation, the RR was 0.10 (95% CI: 0.00–100.16; I^2^ = 95.0%). At 12 months of follow-up, FSG demonstrated a marked, though statistically nonsignificant, reduction in postoperative GERD risk compared with SG (RR = 0.08; 95% CI: 0.01–1.18; I^2^ = 85.1%), suggesting a potentially early protective effect of FSG. By 24 months, this benefit appeared sustained, with a pooled RR of 0.17 (95% CI: 0.03–1.08; I^2^ = 38.6%). The test for subgroup differences was not statistically significant (*p* = 0.66), indicating that the observed effect was stable across follow-up intervals. Therefore, despite the high heterogeneity across studies, all subgroup analyses consistently favored FSG to a lower postoperative GERD burden.

#### 3.4.2. Postoperative GERD Resolution

**Figure 5:**
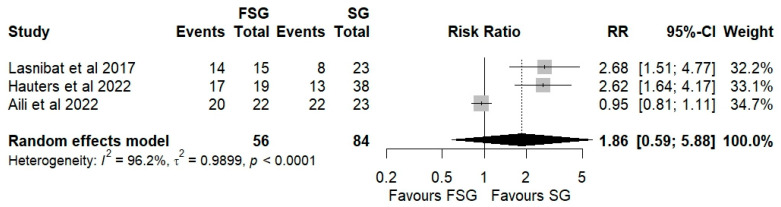
Forest plot showing the risk ratio of postoperative GERD persistence between FSG and SG. Each square represents an individual study’s effect estimate with its 95% confidence interval (horizontal line). The size of the square reflects the study’s weight in the meta-analysis. The dashed vertical line indicates the line of no effect (RR = 1), and the diamond (shadowed area) represents the pooled effect estimate with its 95% confidence interval [9,39,41].

Three studies, including 140 patients, were analyzed to assess postoperative GERD resolution. The pooled results demonstrated a significantly higher likelihood of GERD resolution in the FSG group compared with SG (RR = 1.86; 95% CI: 0.80–4.20; I^2^ = 96.2%; Figure 5). Subgroup analyses stratified by follow-up duration (Figure S4) showed consistent trends. At 12 months of follow-up, the pooled RR was 2.12 (95% CI: 1.45–3.10; I^2^ = 10.5%). At 24 months, the RR was 1.56 (95% CI: 0.39–6.27; I^2^ = 96.9%). The test for subgroup differences was not statistically significant (*p* = 0.68), indicating that the observed effect was stable across follow-up intervals. Although not all subgroup analyses reached statistical significance, the consistent direction of effect supports the conclusion that FSG is associated with greater postoperative GERD resolution compared to SG.

#### 3.4.3. Publication Bias

To assess potential publication bias, the Copas selection model was applied (Table S3). The adjusted effect estimate (−0.44; 95% CI: −1.21 to 0.34) was identical to the unadjusted estimate, and the residual selection bias test (p.rsb = 0.70) indicated no statistically significant bias. These findings suggest that small-study effects or selective publication are unlikely to have influenced the pooled estimate for postoperative GERD outcomes.

### 3.5. Postoperative Complications

**Figure 6:**
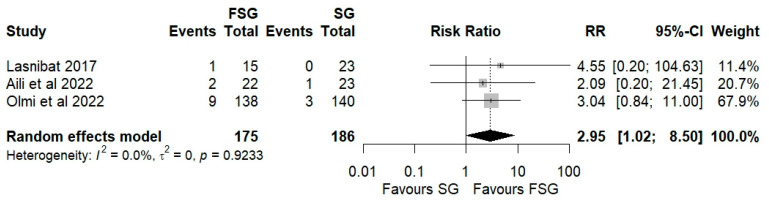
Forest plot showing the risk ratio of postoperative overall (Clavien–Dindo grade ≥ III) complications between FSG and SG. Each square represents an individual study’s effect estimate with its 95% confidence interval (horizontal line). The size of the square reflects the study’s weight in the meta-analysis. The dashed vertical line indicates the line of no effect (RR = 1), and the diamond (shadowed area) represents the pooled effect estimate with its 95% confidence interval [9,39,45].

**Figure 7:**
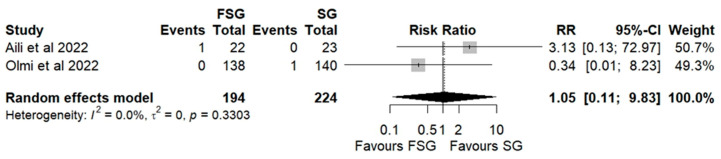
Forest plot showing the risk ratio of postoperative leakage after FSG and SG. Each square represents an individual study’s effect estimate with its 95% confidence interval (horizontal line). The size of the square reflects the study’s weight in the meta-analysis. The dashed vertical line indicates the line of no effect (RR = 1), and the diamond (shadowed area) represents the pooled effect estimate with its 95% confidence interval [9,45].

**Figure 8:**
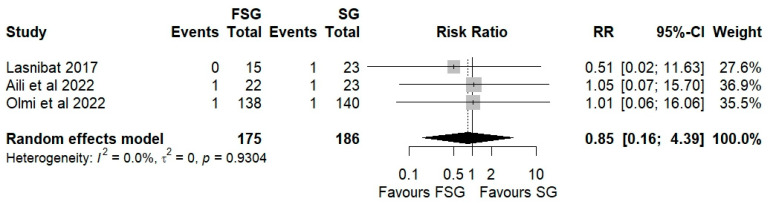
Forest plot showing the risk ratio of postoperative bleeding after FSG and SG. Each square represents an individual study’s effect estimate with its 95% confidence interval (horizontal line). The size of the square reflects the study’s weight in the meta-analysis. The dashed vertical line indicates the line of no effect (RR = 1), and the diamond (shadowed area) represents the pooled effect estimate with its 95% confidence interval [9,39,45].

**Figure 9:**
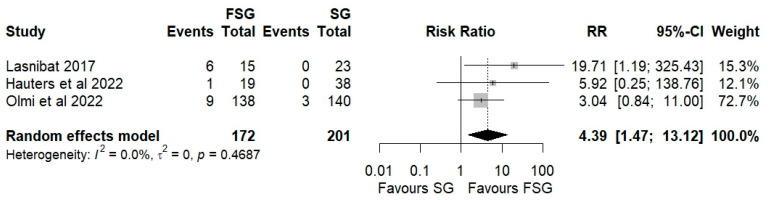
Forest plot showing the risk ratio of reoperation after FSG and SG. Each square represents an individual study’s effect estimate with its 95% confidence interval (horizontal line). The size of the square reflects the study’s weight in the meta-analysis. The dashed vertical line indicates the line of no effect (RR = 1), and the diamond (shadowed area) represents the pooled effect estimate with its 95% confidence interval [39,41,45].

Postoperative complications of FSG following FSG were reported in 4 of 12 studies (280 patients). The pooled analysis showed a significant difference in overall complications (Clavien–Dindo grade ≥ III) between FSG and SG (RR = 2.95; 95% CI: 1.02–8.50; I^2^ = 0%; Figure 6). When stratified by timing (Figure S5), both early (≤30 days) and late (>30 days) complications demonstrated comparable rates across procedures. Specific adverse events—staple line leaks (Figure 7), bleeding (Figure 8), and reoperations (Figure 9)—were analyzed separately. FSG was associated with a significantly higher rate of reoperation (RR = 4.39; 95% CI: 1.47–13.12; I^2^ = 0%, whereas leak (RR = 1.05; 95% CI: 0.11–9.83) and bleeding rates (RR = 0.85; 95% CI: 0.16–4.39) were similar between groups. Detailed wrap-related complications, such as ischemia or migration, were rarely reported and thus not suitable for quantitative synthesis.

To evaluate the potential impact of publication bias, a Copas selection model was applied (Table S4). The adjusted estimate for overall complications (0.33; 95% CI: −0.73 to 1.40) was identical to the unadjusted estimate, with a non-significant test for residual selection bias (*p* = 0.9113) and no between-study heterogeneity (τ^2^ = 0). These findings suggest no evidence of publication bias or residual selection effects, supporting the robustness and reliability of the pooled complication outcomes.

### 3.6. Risk of Bias of Individual Studies

Table 4, Table S5, and Table 5, respectively, display the risk of bias for observational studies and randomized controlled trials. A visual risk-of-bias summary plot (Table 6) was generated to provide an overall view of bias across all included studies and domains. Regarding observational studies, all studies failed to justify the sample size, and none of them ensured that the assessors were blinded to the participants’ level of exposure. Concerning the eighth and tenth questions related to the level and frequency of assessment of the exposure, we considered them not applicable to all studies. Five studies did not have reliable outcome measures or failed to implement them consistently across all participants. Only two studies had a loss to follow-up after baseline higher than 20%. For the rest of the criteria, all studies showed a low risk of bias. Therefore, all studies were classified as good or fair. Regarding the randomized controlled trial, it was considered to have an overall low risk of bias since all domains were classified as having a low risk of bias. Although this systematic review and meta-analysis did not require ethical approval because it analyzed data from previously published studies, all included primary studies reported approval by their respective institutional ethics committees and informed consent from participants, in accordance with the Declaration of Helsinki.

## 4. Discussion

This systematic review and meta-analysis try to synthesize the best available evidence comparing FSG and SG regarding weight loss effectiveness, postoperative GERD outcomes, and safety. This review yielded four principal findings: (1) FSG and SG achieved comparable weight loss outcomes at 12–24 months of follow-up; (2) FSG was associated with a significantly higher rate of GERD resolution and a lower postoperative GERD prevalence; (3) reoperation rates were higher in the FSG group; and (4) overall complication rates (Clavien–Dindo ≥ III) were also higher in FSG.

### 4.1. Effectiveness of FSG

In our review, all of the included FSG studies reported %EWL values in excess of 50% at 12 months. This is consistent with Reinhold’s success threshold, as well as with the outcomes typically reported for SG [47]. Our pooled analysis confirmed that FSG provides comparable weight loss to SG (Hedges’ g = −0.11; 95% CI: −0.99 to 0.76), suggesting that the addition of a fundoplication wrap does not affect metabolic effectiveness. However, substantial heterogeneity (I^2^ = 86%) was observed, likely due to methodological and clinical differences such as surgical calibration, type of wrap, baseline BMI, and variable follow-up durations. To further explore this, exploratory stratified forest plots by follow-up duration were generated, and no evidence of effect modification was found between 12 and 24 months. This indicates that the time to follow up did not account for the observed variability. These findings are consistent with those of a prior meta-analysis by Loo et al. [25], who also reported no significant difference in %EWL (mean difference −0.64, 95% CI: −20.62 to 19.34). In contrast, Mu et al. [48] found a pooled %EWL of 67.8% (95% CI: 55.2–80.5) across seven studies, supporting FSG as a promising bariatric option.

These inconsistencies likely reflect variations in surgical techniques, bougie calibration, patient adherence to dietary regimens, and limited follow-up, reducing the certainty of pooled estimates. Furthermore, the question of whether preserving the gastric fundus impairs weight loss by maintaining ghrelin secretion is still being debated [49]. However, the current evidence does not support this concern. Indeed, several studies suggest that adjunctive fundoplication may even reduce excess weight loss compared with SG alone [25,39,50]. Importantly, our review’s inability to perform subgroup analyses by fundoplication type (Rossetti, Toupet, Nissen) limits interpretation, since the current evidence is heterogeneous and technique-specific. Some evidence suggests that, when constructed properly, %EWL is similar across techniques [39], but robust comparative data remain lacking.

Taken together, these findings reinforce the idea that FSG achieves weight loss comparable to SG, while also highlighting the need for standardized reporting of surgical technique, follow-up duration, and anthropometric outcomes in future comparative studies, to enable more robust quantitative synthesis.

### 4.2. GERD Outcomes

The main disadvantages of SG are the several anatomical and physiological changes leading to reduced lower esophageal sphincter (LES) pressure, alteration of the angle of His, decreased gastric compliance, and delayed gastric emptying [4,6,51]. Maintaining the simple and direct approach of SG, FSG can be a viable alternative to RYGB for preventing late complications and GERD recurrence. It enhances LES tone and decreases esophageal acid exposure by preserving part of the gastric fundus, particularly in the context of Rossetti-Nissen fundoplication involving retroesophageal dissection [52]. Our analysis demonstrated that FSG significantly reduced postoperative GERD prevalence compared with SG (RR = 0.08; 95% CI: 0.01–0.47; *p* < 0.05). This trend was consistent across subgroups defined by GERD ascertainment method and follow-up duration, with moderate heterogeneity (I^2^ = 63.1%). When assessed symptomatically, FSG reduced GERD risk by approximately 67% (RR = 0.33; 95% CI: 0.13–0.81), whereas endoscopy-based evaluations demonstrated a stronger but less precise protective effect (RR = 0.10; 95% CI: 0.00–100.16), likely due to the small number of studies. In addition, FSG was also associated with a higher rate of postoperative GERD resolution (RR = 1.86; 95% CI: 0.80–4.20), with consistent directionality across subgroups. These findings collectively align with a systematic review [48], with a postoperative GERD prevalence reported of just 4.8% following FSG. However, heterogeneity in diagnostic methods—ranging from symptom-based questionnaires to pH monitoring and endoscopy—limits the interpretability of pooled estimates. Future studies should apply standardized GERD definitions, such as those outlined by the Montreal Consensus, and report both symptomatic and objective outcomes to enhance comparability.

### 4.3. Postoperative Complications

The critical drawback of FSG is its increased technical complexity. Fundoplication requires the meticulous handling of the gastric fundus and preservation of vascularization, and errors in these steps can increase perioperative risk (migration, ischemia, breakdown) [18,40]. In fact, our meta-analysis identified a significantly higher rate of overall postoperative complications (Clavien–Dindo ≥ III) with a RR of 2.95 (95% CI: 1.02–8.50; I^2^ = 0%), and reoperations (RR = 4.39; 95% CI: 1.47–13.12; I^2^ = 0%). These findings are reinforced by the publication bias analyses using the Copas selection model, which found no evidence of selective reporting, and are consistent with previous studies [24,44]. These underscores the importance of surgical expertise and appropriate patient selection.

### 4.4. FSG Versus RYGB

Based on IFSO guidelines [45], RYGB remains the procedure of choice for patients with severe obesity, refractory GERD, insulin-dependent type 2 diabetes, or multiple metabolic comorbidities, given its superior long-term outcomes in weight loss and GERD remission [53]. However, though the limited long-term evidence directly comparing FSG and RYGB, FSG may be preferable for patients with obesity and mild-to-moderate GERD or esophagitis who are not suitable candidates for RYGB, particularly those with a complex surgical history, higher perioperative risk, or specific anatomical considerations. In such cases, it may offer improved reflux control compared with SG and achieve similar weight loss, albeit with a higher risk of postoperative complications [54].

### 4.5. Strengths, Limitations, and Certainty of Evidence

Our study has several strengths, particularly in terms of its methodology and analysis. First, it lies in its comprehensive search strategy and adherence to PRISMA and Cochrane standards. Our review expands on previous evidence syntheses through a more comprehensive methodological approach. Compared with Aiolfi et al. [24], whose analysis was limited by a smaller evidence base and shorter search window, our review incorporated studies published up to December 2023. In contrast to Mu et al. [48], who reported pooled weight loss outcomes but included heterogeneous populations and variable follow-up durations, we applied stricter inclusion criteria (BMI ≥ 40 kg/m^2^ or ≥35 kg/m^2^ with comorbidities, minimum 12-month follow-up), ensuring clinical relevance and consistency. Furthermore, while Loo et al. [25] provided an important synthesis of FSG versus SG, our review had a deeper analysis, by standardizing GERD definitions using the Montreal criteria, specifying adverse events such as leak, bleeding, and reoperation separately, and conducting sensitivity and exploratory analyses to visually examine heterogeneity sources based on follow-up time and GERD definition method. Third, though in our meta-analyses, the number of included studies per outcome was limited, we employed the Copas selection model as an alternative approach. This model provides a sensitivity analysis framework for small meta-analyses, exploring how varying degrees of potential publication bias might influence pooled estimates. Finally, we employed dual risk of bias assessments (NIH tool and Cochrane RoB tool), providing a more comprehensive appraisal of study quality compared to the single instruments used in prior reviews. Together, these methodological refinements make this study a more current and clinically informative synthesis of the comparative effectiveness and safety of FSG versus SG.

Nevertheless, several limitations must be acknowledged. First, this review was not prospectively registered in an international database such as PROSPERO. Although a protocol was developed a priori and followed rigorously, the absence of public registration may limit external transparency. Second, given the limited number of eligible studies and the presence of substantial methodological heterogeneity, a formal GRADE assessment of the certainty of evidence was not performed. However, the overall strength of evidence for each outcome was qualitatively evaluated in line with GRADE domains—considering risk of bias, inconsistency, indirectness, and imprecision. The findings should therefore be interpreted as low to moderate certainty, highlighting the need for larger, high-quality randomized trials to strengthen the evidence base on FSG. Third, the small number of studies and their heterogeneous design precluded formal meta-regression and restricted the ability to assess fundoplication-type effects. Fourth, reporting inconsistency—particularly regarding GERD assessment and wrap-specific complications—further constrains interpretation. Some studies relied on symptom questionnaires (e.g., the GERD-Q questionnaire), while others used objective tests, such as pH monitoring or endoscopy. Although GERD can develop or recur years after surgery, many primary studies also lacked standardized follow-up intervals and had short- to mid-term follow-up. Fifth, the overall quality of evidence remains low, primarily because most included studies were observational in design, with moderate to serious risk of bias. The lack of randomized controlled trials means that confounding factors cannot be adequately controlled, limiting the strength of the conclusions drawn. Finally, the limited availability of direct comparisons between FSG and other common GERD-managing bariatric procedures—particularly RYGB—prevents a comprehensive evaluation of the relative effectiveness of FSG in this context [55].

## 5. Clinical Implications

Clinically, FSG appears to provide equivalent weight loss to SG while improving reflux control, offering a potential alternative for patients with obesity and mild-to-moderate GERD who are not ideal candidates for RYGB. However, its higher technical complexity, leading to a higher risk of complications and reoperation, underscores that FSG should be reserved for carefully selected patients and performed in high-volume centers by experienced bariatric surgeons. Standardization of surgical technique, perioperative management, and outcome reporting will be essential to define FSG’s role within the broader bariatric treatment spectrum.

## Figures and Tables

**Table 1:** Data Search Strategy.

**PubMed**	**(“Gastric Sleeve” [All Fields] OR “Sleeve Gastrectomy” [All Fields]) AND (“Fundoplication” [All Fields] OR “Fundoplication” [MeSH Major Topic] OR “Fundoplication” [Title/Abstract] OR “anti-reflux procedures” [All Fields] OR “Nissen-sleeve” [All Fields] OR “N-sleeve” [All Fields] OR “Rossetti” [All Fields] OR “Rossetti Modification” [All Fields] OR “Posterior Fundoplication” [All Fields] OR “Toupet Fundoplication” [All Fields] OR “Dor Fundoplication” [All Fields] OR “Anterior Fundoplication” [All Fields] OR “D-SLEEVE” [All Fields] OR “Collis-Nissen” [All Fields])**
**Scopus**	(ALL (“Gastric sleeve”) OR ALL (“Sleeve gastrectomy”)) AND (ALL (Fundoplication) OR TITLE-ABS (Fundoplication) OR ALL (“anti-reflux procedures”) OR ALL (“Nissen-sleeve”) OR ALL (“N-sleeve”) OR ALL (Rossetti) OR ALL (“Rossetti-Modification”) OR ALL (“Posterior Fundoplication”) OR ALL (“Toupet Fundoplication”) OR ALL (“Dor Fundoplication”) OR ALL (“Anterior Fundoplication”) OR ALL (“D-sleeve”) OR ALL (“Collis-Nissen”))
**Web of Science**	(ALL = (Gastric sleeve) OR ALL = (Sleeve Gastrectomy)) AND (ALL = (Fundoplication) OR TI = (Fundoplication) OR AB = (Fundoplication) OR ALL = (anti-reflux procedures) OR ALL = (Nissen-sleeve) OR ALL = (N-sleeve) OR ALL = (Rossetti) OR ALL = (Rossetti Modification) OR ALL = (Posterior Fundoplication) OR ALL = (Toupet Fundoplication) OR ALL = (Dor Fundoplication) OR ALL = (Anterior Fundoplication) OR ALL = (D-SLEEVE) OR ALL = (Collis-Nissen))

**Table 2:** Study characteristics.

	Study Design	No. Patients	Female (%)	Age (Years) (Mean ± SD)	BMI (kg/m^2^)	Procedure	Operative Time (min) (Mean ± SD)	HLOS (Days) (Mean ± SD)	Follow-Up (Months)
Hawasli et al., 2016 [38]	RS	40	31 (78%)	-	49 ± 8	Dor-Sleeve	84 ± 20	1.6 ± 0.9	24
Nocca et al., 2016 [18]	PS	25	13 (54%)	41	42 (35–23)	Nissen-Sleeve	84	5	12
Lasnibat et al., 2017 [39]	RS	15 (A) 23 (B)	14 (93%) (A) 12 (52%) (B)	46.2 (A) 37.3 (B)	33.9 ± 2.1 (A) 37.5 ± 4.4 (B)	Nissen-Sleeve Sleeve Gastrectomy	157 ± 22.13 (A) 87 ± 15 (B)	4.6 (A) 2.6 (B)	12
Olmi et al., 2017 [40]	RS	40	34 (75%)	39.6	44.4 ± 4.7	Rossetti-Sleeve	38	4	12
Del Genio et al., 2020 [11]	PS	32 (A) 25 (B)	20 (63%) (A)	38(A)	46 (37–52) (A) 46.1 (38–58) (B)	Dor-Sleeve Sleeve Gastrectomy	-	-	14
Amor et al., 2020 [43]	PS	70	56 (80%)	42	40 (35–60)	Nissen-Sleeve	62	2	12
Carandina et al., 2020 [42]	RS	28	25 (88%)	40 ± 10	41.2 ± 5.5	Nissen-Sleeve	70 ± 12.7	2.9 ± 0.7	12
Olmi et al., 2021 [19]	PS	58	42 (72%)	43 ± 8.4	41.9 ± 4.6 (B)	Rossetti-Sleeve	51.8 ± 19.5	-	12
Aili et al., 2022 [9]	RS	22 (A) 23 (B)	17 (77%) (A) 17 (74%) (B)	37.9(A) 36.3 (B)	38.4 (31.1–50.2) (A) 39.6 (33.4–50.8) (B)	Fundoplication-Sleeve Sleeve Gastrectomy	6.5 (A) 7.3 (B)	6.0 (A) 7.3 (B)	22
Hauters et al., 2023 [41]	RS	19 (A) 38 (B)	14 (74%) (A) 21 (55%) (B)	42 ± 15 (A), 40 ± 17 (B)	43 ± 5 (A), 42 ± 5 (B)	Toupet-Sleeve Sleeve Gastrectomy	89 ± 18 (A) 68 ± 12 (B)	2 (A), 2 (B)	108
Olmi et al., 2022 [45]	RCT	138 (A) 140 (B)	108 (78%) (A) 100 (71%) (B)	40.8 ± 11.1 (A) 41.3 ± 9.8 (B)	43.4 ± 5.9 (A) 45.2 ± 7 (B)	Rosetti-Sleeve Sleeve Gastrectomy	47.4 ± 17.4 (A) 48.4 ± 15.1 (B)	3.9 ± 4 (A), 3.1 ± 0.5 (B)	12
Nocca et al., 2022 [44]	PS	365	277 (76%)	41.2 ± 14.1	41.6 ± 5.4	Nissen-Sleeve	83	3.4	12

PS—Prospective Study; RS—Retrospective Study; RCT—Randomized Controlled Trial; A—Group A Patients submitted to fundoplication sleeve gastrectomy; B—Group B Patients submitted to sleeve gastrectomy; BMI—Body Mass Index; HLOS—hospital length of stay.

**Table 3:** Weight loss assessment after FSG in the studies included in the qualitative synthesis.

Author, Year	%EWL	%EBMIL	%TWL
Hawasli et al., 2016 [38]	-	69 ± 27%	-
Nocca et al., 2016 [18]	58 ± 23%	-	27 ± 10%
Lasnibat et al., 2017 [39]	82.02%	-	-
Olmi et al., 2017 [40]	61.7 ± 13.6%	73.3 ± 9.9%	-
Del Genio et al., 2020 [11]	59%	-	-
Amor et al., 2020 [43]	69 ± 20%	-	25 ± 8%
Carandina et al., 2020 [42]	-	-	-
Olmi et al., 2021 [19]	-	-	-
Aili et al., 2022 [9]	83.3 ± 28.5%	-	31.2 ± 8%
Hauters et al., 2023 [41]	61 ± 23%	-	-
Olmi et al., 2022 [45]	-	-	32.2 ± 7.6%
Nocca et al., 2022 [44]	77.3 ± 26.3%	-	-

**Table 4:** Detailed results of the risk of bias assessments for included observational studies regarding the comparison of the morbidity and mortality of type of fundoplication sleeve gastrectomy.

Author, Year	1	2	3	4	5	6	7	8	9	10	11	12	13	14	Quality Rating
Hawasli et al., 2016 [38]	Yes	Yes	Yes	Yes	No	Yes	Yes	NA	Yes	NA	No	No	No	Yes	Fair
Nocca et al., 2016 [18]	Yes	Yes	Yes	Yes	No	Yes	Yes	NA	Yes	NA	No	No	Yes	Yes	Fair
Lasnibat et al., 2017 [39]	Yes	Yes	Yes	Yes	No	Yes	Yes	NA	Yes	NA	Yes	No	Yes	Yes	Good
Olmi et al., 2017 [40]	Yes	Yes	Yes	Yes	No	Yes	Yes	NA	Yes	NA	Yes	No	Yes	Yes	Good
Del Genio et al., 2020 [11]	Yes	Yes	Yes	Yes	No	Yes	Yes	NA	Yes	NA	Yes	No	Yes	Yes	Good
Amor et al., 2020 [43]	Yes	Yes	Yes	Yes	No	Yes	Yes	NA	Yes	NA	Yes	No	Yes	Yes	Good
Carandina et al., 2020 [42]	Yes	Yes	Yes	Yes	No	Yes	Yes	NA	Yes	NA	No	No	Yes	Yes	Fair
Olmi et al., 2021 [19]	Yes	Yes	Yes	Yes	No	Yes	Yes	NA	Yes	NA	No	No	Yes	Yes	Fair
Aili et al., 2022 [9]	Yes	Yes	Yes	Yes	No	Yes	Yes	NA	Yes	NA	Yes	No	Yes	Yes	Good
Hauters et al., 2023 [41]	Yes	Yes	Yes	Yes	No	Yes	Yes	NA	Yes	NA	Yes	No	No	Yes	Fair
Nocca et al., 2022 [44]	Yes	Yes	Yes	Yes	No	Yes	Yes	NA	Yes	NA	No	No	Yes	Yes	Fair

NA: non-applicable. Risk of bias assessments are based on National Institutes of Health quality (NIH) assessment criteria for observational studies—this tool consists of a form with 14 Y (yes) or N (no) questions (related to the research question, study population, exposure, outcome, blinding, follow-up, and statistical analyses) and a final quality rating—G (good), F (fair), P (poor), classifying the study according to its potential risk of bias.

**Table 5:** Detailed results of the risk of bias assessments for the randomized controlled trial.

Author, Year	D1	D2	D3	D4	D5	Overall
Olmi et al., 2022 [45]	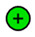	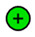	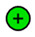	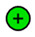	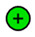	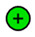
	D1: Bias arising from the randomization process; D2: Bias due to deviation from intended intervention; D3: Bias due to missing outcome data; D4: Bias in measurement of the outcome; D5: Bias in selection of the reported result Judgement: 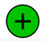 Low					

**Table 6:** Detailed results of the overall view of bias across all included studies and domains.

Author, Year	D1	D2	D3	D4	Overall
Hawasli et al., 2016 [38]	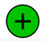	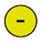	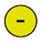	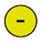	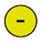
Nocca et al., 2016 [18]	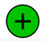	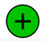	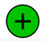	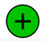	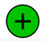
Lasnibat et al., 2017 [39]	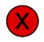	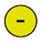	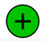	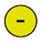	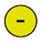
Olmi et al., 2017 [40]	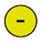	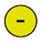	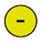	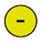	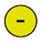
Del Genio et al., 2020 [11]	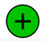	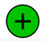	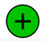	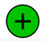	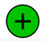
Amor et al., 2020 [43]	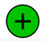	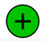	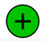	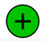	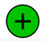
Carandina et al., 2020 [42]	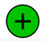	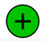	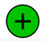	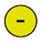	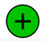
Olmi et al., 2021 [19]	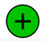	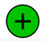	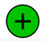	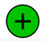	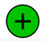
Aili et al., 2022 [9]	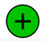	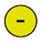	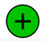	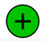	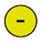
Hauters et al., 2023 [41]	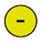	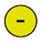	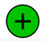	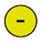	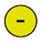
Nocca et al., 2022 [44]	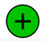	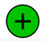	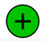	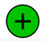	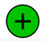
Olmi et al., 2022 [45]	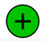	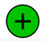	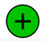	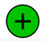	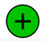
	D1: Bias due to deviation from intended intervention; D2: Bias due to missing data; D3: Bias in measurement of the outcome; D4: Bias in selection of the reported result Judgement: 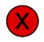 High 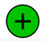 Low 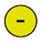 Incertain				

## Data Availability

The original contributions presented in this study are included in the article/Supplementary Materials. Further inquiries can be directed to the corresponding author.

## Supplementary Materials

The following supporting information can be downloaded at: https://www.mdpi.com/article/10.3390/jcm14217723/s1, Figure S1: Forest plot showing the pooled standardized mean difference of %EWL between FSG and SG and its subgroup analysis by follow-up duration; Figure S2: Forest plot showing the risk ratio of postoperative GERD’s prevalence between FSG and SG and its subgroup analysis by GERD assessment method; Figure S3: Forest plot showing the risk ratio of postoperative GERD’s prevalence between FSG and SG and its subgroup analysis by follow-up duration; Figure S4: Forest plot showing the risk ratio of postoperative GERD persistence between FSG and SG and its subgroup analysis by follow-up duration; Figure S5: Forest plot showing the risk of postoperative overall (Clavien–Dindo grade ≥ III) complications between FSG and SG, and its subgroup analysis based on their timing; Table S1: PRISMA Checklist; Table S2: Assessment of potential publication bias by Copas’ selection model showing the outcome of %EWL between FSG and SG; Table S3: Assessment of potential publication bias by Copas’ selection model showing the outcome of GERD prevalence between FSG and SG; Table S4: Assessment of potential publication bias by Copas’ selection model showing the postoperative overall complication between FSG and SG; Table S5: Detailed results of the risk of bias assessments for the non-randomized controlled trial.

## Abbreviations

The following abbreviations are used in this manuscript:

SG	Laparoscopic sleeve gastrectomy
GERD	Gastroesophageal reflux disease (GERD)
FSG	Fundoplication sleeve gastrectomy
%EWL	Percentage of excess Weight Loss
%TWL	Percentage of total weight loss
RYGB	Roux-en-Y Gastric Bypass
BMI	Body Mass Index
F.A.C	Filipe Amorim Cruz
D.F.L	Diogo Fernandes Lopes
