# Supplementary material for: Efficacy and Safety of Fundoplication Sleeve Gastrectomy in Obesity and GERD: A Systematic Review and Meta-Analysis
Source: J Clin Med. 2025 Oct 30;14(21):7723. doi: 10.3390/jcm14217723 (PMC12610764; doi:10.3390/jcm14217723)
Supplement: Supplementary file 1 [file jcm-14-07723-s001.zip › jcm-3888737-supplementary.pdf]

# Supplementary Materials

**Protocol for Systematic Review and Meta-Analysis: Effectiveness, Safety, and Feasibility of Fundoplication Sleeve Gastrectomy (FSG) versus Standard Sleeve Gastrectomy (SG) in Patients with Severe Obesity**

**Date created:** September 2023

**Authors:** F.A.C., D.F.L., B.S.S, H.S.S.,

**Version:** 1.0 (finalized before database search and data extraction)

**Objective:** To systematically review and quantitatively synthesize the available evidence on the clinical effectiveness, safety, and feasibility of FSG compared with standard SG in adults with severe obesity.

**Primary outcomes:**

- Postoperative gastroesophageal reflux disease (GERD) prevalence and resolution
- Weight loss outcomes (%EWL, %TWL, %EBMIL)
- Postoperative overall complications (Clavien–Dindo grade ≥ III)

**Secondary outcomes:**

- Reoperation rates
- Early vs. late postoperative complications
- Procedure-specific adverse events (leak, bleeding)

**Eligibility criteria:** Original studies including adults (BMI ≥ 35 kg/m²) undergoing FSG vs. SG; minimum follow-up 12 months. The exclusion criteria comprised studies with a follow-up duration of less than 1 year, studies that were reviews, meta-analyses, case reports, technical reports, editorials, letters to the editor, and animal studies.

**Databases searched:** PubMed, Scopus, Web of Science (up to December 31, 2023).

**Search strategy:**

| | |
|---|---|
| **PubMed** | ("Gastric Sleeve"[All Fields] OR "Sleeve Gastrectomy"[All Fields]) AND (“Fundoplication”[All Fields] OR “Fundoplication”[MeSH Major Topic] OR “Fundoplication”[Title/Abstract] OR “anti-reflux procedures”[All Fields] OR “Nissen-sleeve”[All Fields] OR “N-sleeve”[All Fields] OR “Rossetti”[All Fields] OR “Rossetti Modification”[All Fields] OR “Posterior Fundoplication”[All Fields] OR “Toupet Fundoplication”[All Fields] OR “Dor Fundoplication”[All Fields] OR “Anterior Fundoplication”[All Fields] OR “D-SLEEVE”[All Fields] OR “Collis-Nissen”[All Fields]) |

| | |
|---|---|
| **Scopus** | (ALL ("Gastric sleeve") OR ALL ("Sleeve gastrectomy")) AND (ALL (Fundoplication) OR TITLE-ABS (Fundoplication) OR ALL ("anti-reflux procedures") OR ALL ("Nissen-sleeve") OR ALL ("N-sleeve") OR ALL (Rossetti) OR ALL ("Rossetti-Modification") OR ALL ("Posterior Fundoplication") OR ALL ("Toupet Fundoplication") OR ALL ("Dor Fundoplication") OR ALL ("Anterior Fundoplication") OR ALL ("D-sleeve") OR ALL ("Collis-Nissen")) |
| **Web of Science** | (ALL=(Gastric sleeve) OR ALL=(Sleeve Gastrectomy)) AND (ALL=(Fundoplication) OR TI=(Fundoplication) OR AB=(Fundoplication) OR ALL=(anti-reflux procedures) OR ALL=(Nissen-sleeve) OR ALL=(N-sleeve) OR ALL=(Rossetti) OR ALL=(Rossetti Modification) OR ALL=(Posterior Fundoplication) OR ALL=(Toupet Fundoplication) OR ALL=(Dor Fundoplication) OR ALL=(Anterior Fundoplication) OR ALL=(D-SLEEVE) OR ALL=(Collis-Nissen)) |

**Screening and data extraction:** Independently by two reviewers, with disagreements resolved by a third reviewer

**Quality assessment:** NIH Quality Assessment Tool for observational studies and Cochrane RoB tool for randomized trials.

**Statistical analysis:** Random-effects meta-analysis (DerSimonian–Laird); heterogeneity assessed with $I^2$; leave-one-out sensitivity analysis; stratified exploratory analyses by follow-up time and GERD assessment method.

**Software:** R (version 4.4.1), *meta* package.

## Figures and Tables

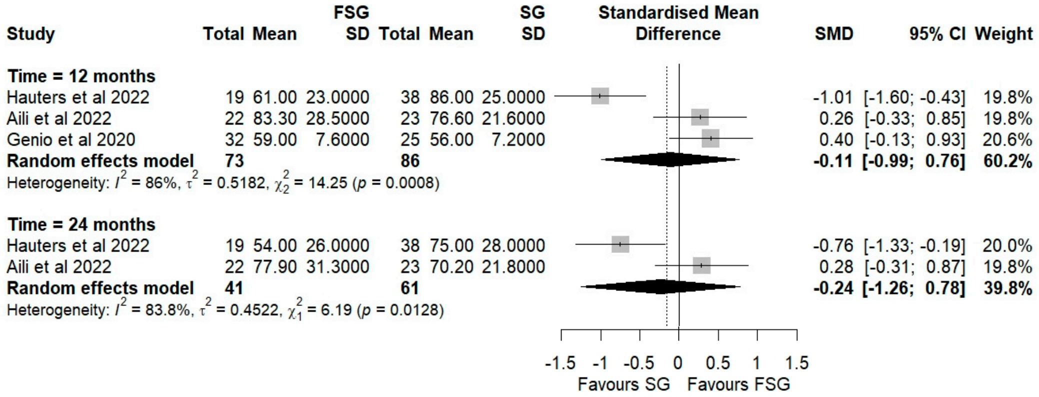


**Figure S1** – Forest plot showing the pooled standardized mean difference of %EWL between FSG and SG and its subgroup analysis by follow-up duration

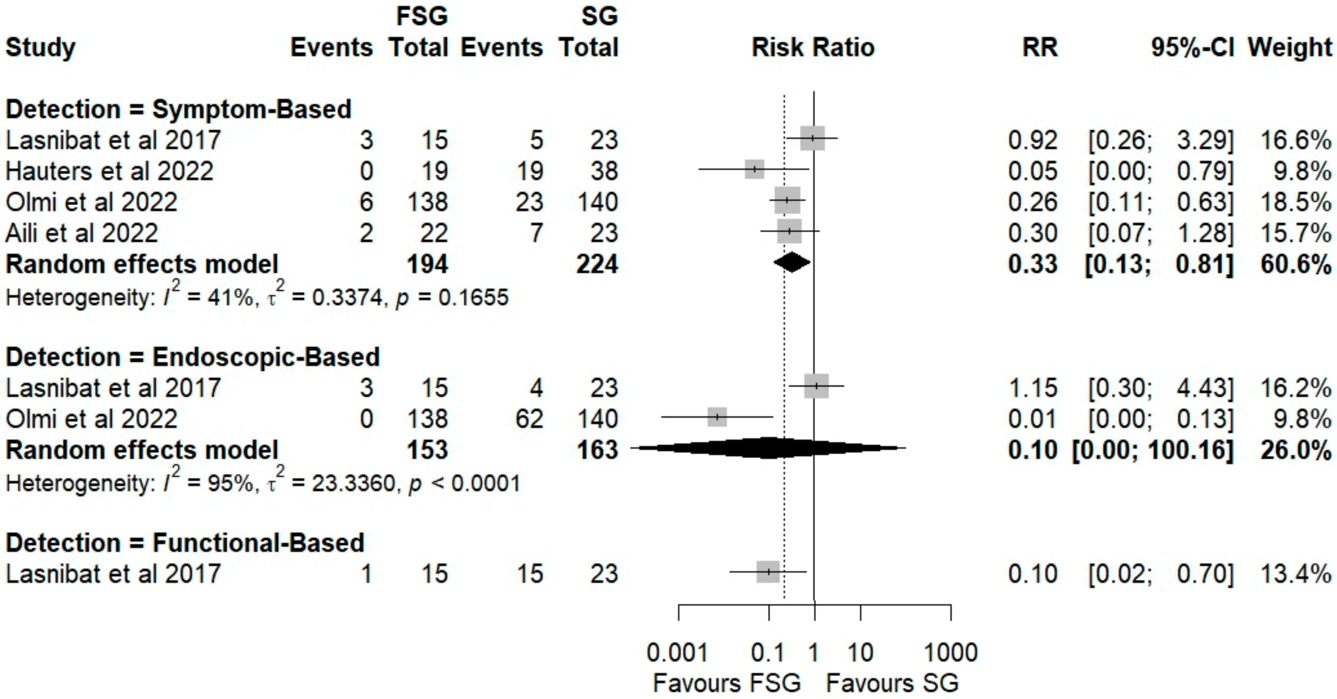


**Figure S2** – Forest plot showing the risk ratio of postoperative GERD’s prevalence between FSG and SG and its subgroup analysis by GERD assessment method

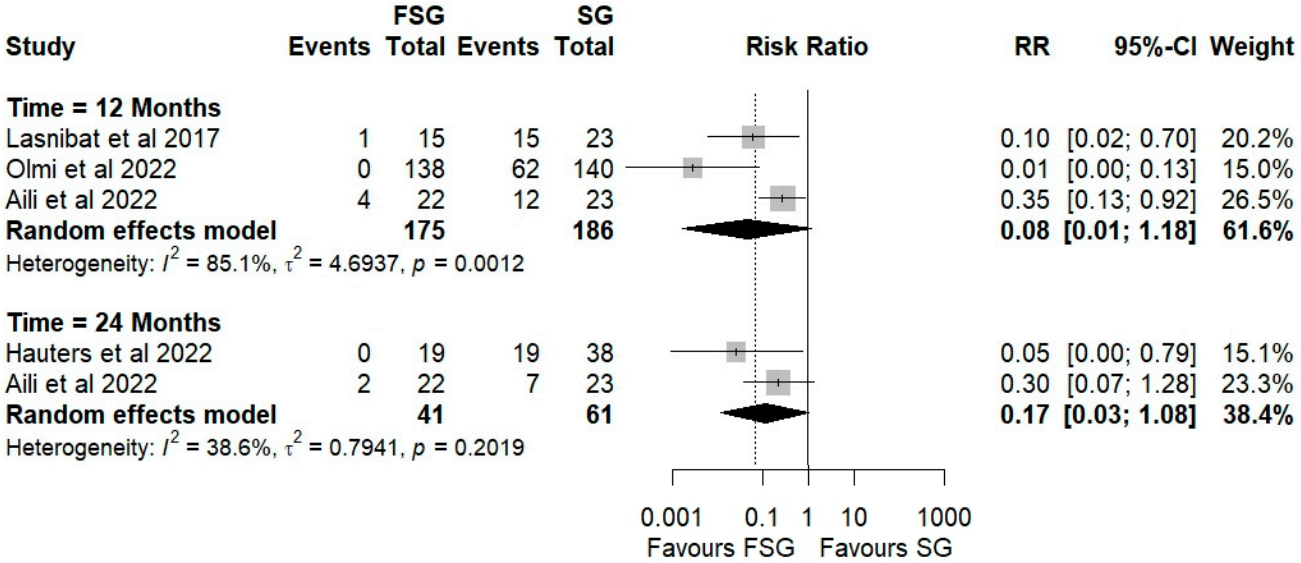


**Figure S3** – Forest plot showing the risk ratio of postoperative GERD's prevalence between FSG and SG and its subgroup analysis by follow-up duration

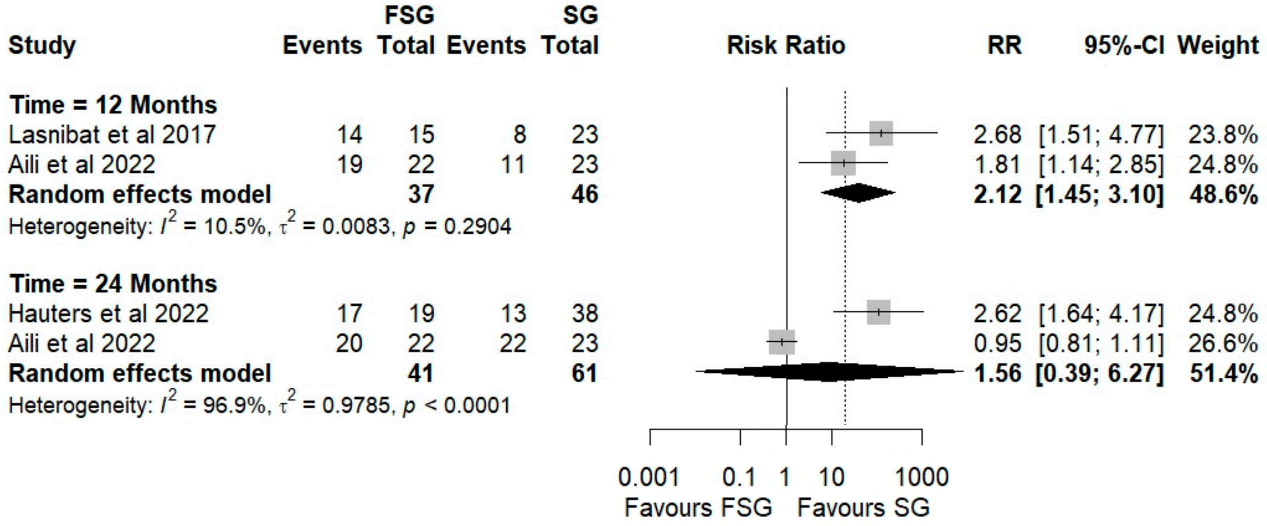


**Figure S4** – Forest plot showing the risk ratio of postoperative GERD persistence between FSG and SG and its subgroup analysis by follow-up duration

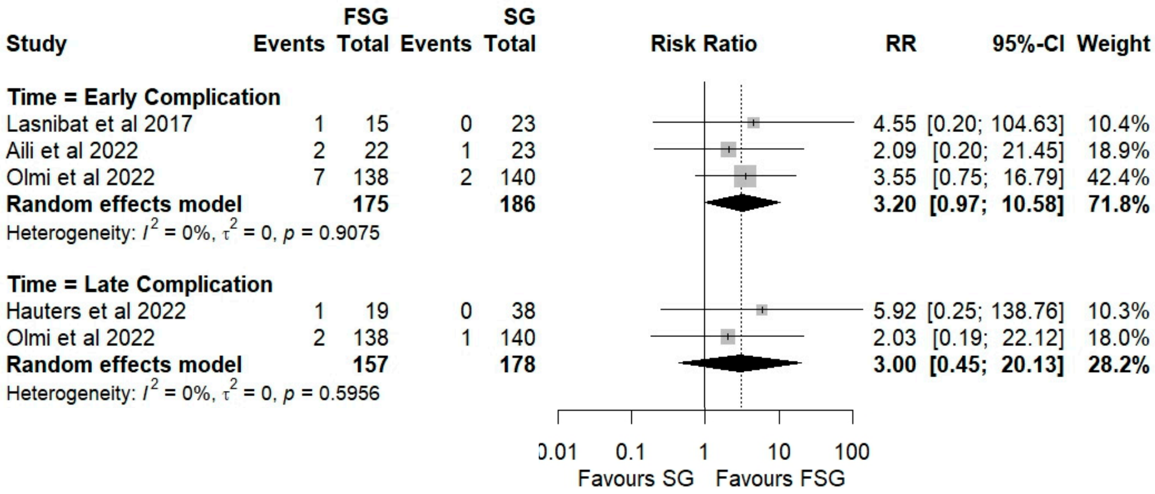


**Figure S5 –** Forest plot showing the risk of postoperative overall (Clavien–Dindo grade ≥ III) complications between FSG and SG, and its subgroup analysis based on their timing

**Table S1** – PRISMA Checklist

| Section and Topic | Item # | Checklist item | Location where item is reported |
|---|---|---|---|
| **TITLE** | | | |
| Title | 1 | Identify the report as a systematic review. | Page 1 |
| **ABSTRACT** | | | |
| Abstract | 2 | See the PRISMA 2020 for Abstracts checklist. | Page 1 |
| **INTRODUCTION** | | | |
| Rationale | 3 | Describe the rationale for the review in the context of existing knowledge. | Page 2&3 – line 45-99 |
| Objectives | 4 | Provide an explicit statement of the objective(s) or question(s) the review addresses. | Page 2 – Line 100-102 |
| **METHODS** | | | |
| Eligibility criteria | 5 | Specify the inclusion and exclusion criteria for the review and how studies were grouped for the syntheses. | Page 4 – Line 119-125 |
| Information sources | 6 | Specify all databases, registers, websites, organisations, reference lists and other sources searched or consulted to identify studies. Specify the date when each source was last searched or consulted. | Page 4 – line 112-115 |
| Search strategy | 7 | Present the full search strategies for all databases, registers and websites, including any filters and limits used. | Page 4 – line 112-113 + Table 1 |
| Selection process | 8 | Specify the methods used to decide whether a study met the inclusion criteria of the review, including how many reviewers screened each record and each report retrieved, whether they worked independently, and if applicable, details of automation tools used in the process. | Page 4 – line 117-125 + Figure 1 |
| Data collection process | 9 | Specify the methods used to collect data from reports, including how many reviewers collected data from each report, whether they worked independently, any processes for obtaining or confirming | Page 4 – line 114-115; line 126-128 |

| Section and Topic | Item # | Checklist item | Location where item is reported |
|---|---|---|---|
| | | data from study investigators, and if applicable, details of automation tools used in the process. | |
| Data items | 10a | List and define all outcomes for which data were sought. Specify whether all results that were compatible with each outcome domain in each study were sought (e.g. for all measures, time points, analyses), and if not, the methods used to decide which results to collect. | Page 4 – line 121-122; line 128-148 |
| | 10b | List and define all other variables for which data were sought (e.g. participant and intervention characteristics, funding sources). Describe any assumptions made about any missing or unclear information. | Page 4 – Line 128-131 |
| Study risk of bias assessment | 11 | Specify the methods used to assess risk of bias in the included studies, including details of the tool(s) used, how many reviewers assessed each study and whether they worked independently, and if applicable, details of automation tools used in the process. | Page 5 – Line 149-158 |
| Effect measures | 12 | Specify for each outcome the effect measure(s) (e.g. risk ratio, mean difference) used in the synthesis or presentation of results. | Page 5 – Line 159-165 |
| Synthesis methods | 13a | Describe the processes used to decide which studies were eligible for each synthesis (e.g. tabulating the study intervention characteristics and comparing against the planned groups for each synthesis (item #5)). | Page 4 – Line 119-125 |
| | 13b | Describe any methods required to prepare the data for presentation or synthesis, such as handling of missing summary statistics, or data conversions. | Page 5 – Line 169-174; Line 179-181; Page 6 – Page 189-192 |
| | 13c | Describe any methods used to tabulate or visually display results of individual studies and syntheses. | Results of individual studies were tabulated in structured tables (e.g., study characteristics, outcomes, and risk of bias assessments and visually displayed using forest plots to illustrate effect sizes, confidence intervals, and pooled estimates, alongside measures of heterogeneity ($I^2$). Narrative synthesis further complemented these visual and tabular summaries.” |
| | 13d | Describe any methods used to synthesize results and provide a rationale for the choice(s). If meta-analysis was performed, describe the model(s), method(s) to identify the presence and extent of statistical heterogeneity, and software package(s) used. | Page 5 - Line 174 - 178 |
| | 13e | Describe any methods used to explore possible causes of heterogeneity among study results (e.g. subgroup analysis, meta-regression). | Page 5 – Line 178-188 |
| | 13f | Describe any sensitivity analyses conducted to assess robustness | Page 5 – Line 178- |

| Section and Topic | Item # | Checklist item | Location where item is reported |
|---|---|---|---|
| | | of the synthesized results. | 179, 179-181, Page 6 – line 191-193 |
| Reporting bias assessment | 14 | Describe any methods used to assess risk of bias due to missing results in a synthesis (arising from reporting biases). | Page 6 – Line 189-193 |
| Certainty assessment | 15 | Describe any methods used to assess certainty (or confidence) in the body of evidence for an outcome. | Page 5 – Line 150-158 |
| **RESULTS** | | | |
| Study selection | 16a | Describe the results of the search and selection process, from the number of records identified in the search to the number of studies included in the review, ideally using a flow diagram. | Page 6 - Line 198-204 |
| | 16b | Cite studies that might appear to meet the inclusion criteria, but which were excluded, and explain why they were excluded. | Figure 1 |
| Study characteristics | 17 | Cite each included study and present its characteristics. | Line 207-214 + Table 2 |
| Risk of bias in studies | 18 | Present assessments of risk of bias for each included study. | Page 14 – 3.6 Risk of Bias of Individual Studies Section + Table 4,5, 6 and A4 |
| Results of individual studies | 19 | For all outcomes, present, for each study: (a) summary statistics for each group (where appropriate) and (b) an effect estimate and its precision (e.g. confidence/credible interval), ideally using structured tables or plots. | Table 2<br>For % EWL: Page 9 + Table 3 + Figure 2-3<br>Postoperative GERD: Page 11-12+ Figure 4-5<br>Postoperative complications: Page 13-14 + figure 6-9 |
| Results of syntheses | 20a | For each synthesis, briefly summarise the characteristics and risk of bias among contributing studies. | For % EWL: Page 9 + Table 3 + Figure 2-3<br>Postoperative GERD: Page 11-12+ Figure 4-5<br>Postoperative complications: Page 13-14 + figure 6-9 |
| | 20b | Present results of all statistical syntheses conducted. If meta-analysis was done, present for each the summary estimate and its precision (e.g. confidence/credible interval) and measures of statistical heterogeneity. If comparing groups, describe the direction of the effect. | Page 9-14, Results section:<br>The paper reports pooled summary estimates (odds ratios / relative risks) with 95% confidence intervals, and also provides I² and p-values for heterogeneity.<br><br>Figures (forest plots):<br>Forest plots visually |

| Section and Topic | Item # | Checklist item | Location where item is reported |
|---|---|---|---|
| | | | display the direction of effects, summary estimates, CIs, and heterogeneity measures.<br><br>Figures 2-9: Contain structured presentation of effect estimates and confidence intervals per study, followed by pooled estimates. |
| | 20c | Present results of all investigations of possible causes of heterogeneity among study results. | Figure A1-A5 |
| | 20d | Present results of all sensitivity analyses conducted to assess the robustness of the synthesized results. | Figure A1-A5 + Table A1-A3 |
| Reporting biases | 21 | Present assessments of risk of bias due to missing results (arising from reporting biases) for each synthesis assessed. | Page 14 – 3.6 .Risk of Bias of Individual Studies + Table 4, A4, and 5 |
| Certainty of evidence | 22 | Present assessments of certainty (or confidence) in the body of evidence for each outcome assessed. | Table 4-6, A4 |
| **DISCUSSION** | | | |
| Discussion | 23a | Provide a general interpretation of the results in the context of other evidence. | Page 16 – 4. Discussion |
| | 23b | Discuss any limitations of the evidence included in the review. | Page 20 – “4.5 Strengths, Limitations, and Certainty of Evidence – Third, the small number of studies and their heterogeneous design (…) prevents a comprehensive evaluation of the relative effectiveness of FSG in this context” |
| | 23c | Discuss any limitations of the review processes used. | Page 19-20 – “4.5 Strengths, Limitations, and Certainty of Evidence – “First, this review was not prospectively registered in an international database such as PROSPERO. Although a protocol was developed a priori and followed |

| Section and Topic | Item # | Checklist item | Location where item is reported |
|---|---|---|---|
| | | | rigorously, the absence of public registration may limit external transparency (…)Third, the small number of studies and their heterogeneous design precluded formal meta-regression and restricted the ability to assess fundoplication-type effects."<br>"Fourth, reporting inconsistency—particularly regarding GERD assessment and wrap-specific complications—further constrains interpretation."" |
| | 23d | Discuss implications of the results for practice, policy, and future research. | Page 18 – 4. Discussion (4.4 - FSG vs SG vs RYGB paragraph + Conclusion) ;<br>Page 20 - 5. Clinical Implications – "(…)Standardization of surgical technique, perioperative management, and outcome reporting will be essential to define FSG's role within the broader bariatric treatment spectrum." |
| **OTHER INFORMATION** | | | |
| Registration and protocol | 24a | Provide registration information for the review, including the register name and registration number, or state that the review was not registered. | Page 4 – Line 107 - 111 |
| | 24b | Indicate where the review protocol can be accessed, or state that a protocol was not prepared. | Page 4 – Line 110-111: "(…)(protocol developed and 110 timestamped in September 2023; see Supplementary Material)." |
| | 24c | Describe and explain any amendments to information provided at registration or in the protocol. | Page 4 – Line 108-110 |
| Support | 25 | Describe sources of financial or non-financial support for the | Page 20 - Funding: |

| Section and Topic | Item # | Checklist item | Location where item is reported |
|---|---|---|---|
| | | review, and the role of the funders or sponsors in the review. | This research received no external funding |
| Competing interests | 26 | Declare any competing interests of review authors. | Page 20 - Conflict of Interest: The authors declare no conflict of interest or financial support concerning the research, authorship, and publication of this article. |
| Availability of data, code and other materials | 27 | Report which of the following are publicly available and where they can be found: template data collection forms; data extracted from included studies; data used for all analyses; analytic code; any other materials used in the review. | Supplementary Material – Page 12 - **Data and Code Availability:** The template data extraction form, extracted datasets, and analytic R code used for the meta-analyses are available in the Supplementary Materials. All data were derived from published studies cited in this review. The PRISMA 2020 checklist and protocol (timestamped September 2023) are also provided as supplementary files to ensure transparency and reproducibility |

**Table S2** – Assessment of potential publication bias by Copas' selection model showing the outcome of %EWL between FSG and SG

| Author | Year | Copas Model | 95% CI | Estimated rho |
|---|---|---|---|---|
| **Hauters et al** | 2022 | | | |
| **Genio et al** | 2020 | -0.11 | [-0.98; 0.76] | 0.29 |
| **Aili et al** | 2022 | | | |

**Table S3** – Assessment of potential publication bias by Copas' selection model showing the outcome of GERD prevalence between FSG and SG

| Author | Year | Copas Model | 95% CI | Estimated rho |
|---|---|---|---|---|
| **Lasnibat** | 2017 | | | |
| **Aili et al** | 2022 | -0.44 | [ -1.21; 0.34] | 0.70 |
| **Hauters** | 2022 | | | |

**Table S4** – Assessment of potential publication bias by Copas' selection model showing the postoperative overall complication between FSG and SG

| Author | Year | Copas Model | 95% CI | Estimated rho |
|---|---|---|---|---|
| **Lasnibat** | 2017 | | | |
| **Aili et al** | 2022 | 0.33 | [-0.73; 1.40] | 0.91 |
| **Olmi et al** | 2022 | | | |

**Table S5:** Detailed results of the risk of bias assessments for the non-randomized controlled trial

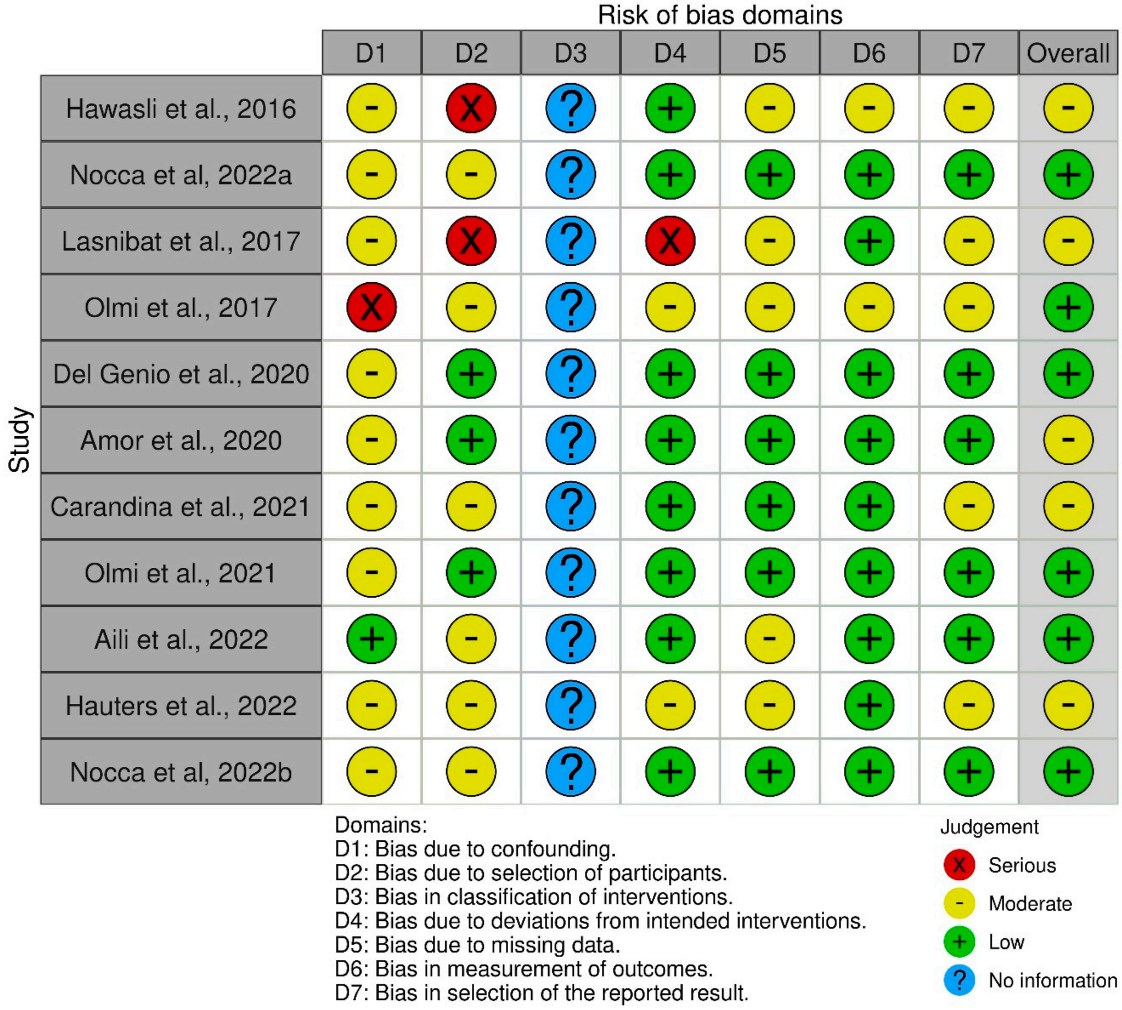

**Data and Code Availability:**

The template data extraction form, extracted datasets, and analytic R code used for the meta-analyses are available in the Supplementary Materials. All data were derived from published studies cited in this review. The PRISMA 2020 checklist and protocol (timestamped September 2023) are also provided as supplementary files to ensure transparency and reproducibility
